# Effectiveness of auricular acupressure on constipation and related quality of life among the older people in the residential care home: a randomized clinical trial

**DOI:** 10.1186/s12877-023-03881-7

**Published:** 2023-03-27

**Authors:** Mahdi Aminizadeh, Batool Tirgari, Omsalimeh Roudi Rashtabadi, Yunes Jahani, Haleh Tajadini

**Affiliations:** 1 Department of Medical Surgical Nursing, Razi Faculty of Nursing and Midwifery, Kerman University of Medical Sciences, Kerman, Iran; 2 Nursing Research Center, Kerman University of Medical Sciences, Kerman, Iran; 3 Modelling in Health Research Center, Institute for Futures Studies in Health, Kerman University of Medical Sciences, Kerman, Iran; 4 Neuroscience Research Center, Institute of Neuropharmacology, Kerman University of Medical Sciences, Kerman, Iran

**Keywords:** Auricular acupressure, Constipation, Elderly, Quality of life

## Abstract

**Introduction:**

Constipation can be one of the biggest health problems for the older people that has negative effects on their quality of life. Some studies have reported that new non-pharmacological interventions such auricular acupressure have promising results in the management of constipation. This study was performed to investigate the effect of auricular acupressure on constipation and health-related quality of life in the older people living in the residential care home.

**Methods:**

Sample of this randomized clinical trial consisted of 53 older people with chronic constipation living in a residential care home in the southeast of Iran (Kerman city). The participants were randomly assigned to intervention (*n* = 27) and control (*n* = 26) groups. Auricular acupressure was applied to seven auricular acupoints (large intestine, rectum, San Jiao, spleen, lung, sympathetic, and subcortex) using Vaccaria seeds for the intervention group and for the control group, seedless auricular plasters were used at the seven auricular acupoints for 10 days. Data were collected before the intervention, end of the intervention, and 10-day follow-up using demographic and clinical, Patient Assessment of Constipation-Symptom, and Patient Assessment of Constipation-Quality of Life questionnaires. The SPSS-22 software was used for data analysis.

**Results:**

The difference between groups and times was significant in constipation and related quality of life and scores. The mean score of constipation at the end of intervention was 0.41 less in the intervention group than the control group (*P* < 0.0001). This mean score, in the intervention group also on the 10-day follow-up was 0.09 less than the control group (*P* = 0.004), which indicates a decrease in the severity of constipation symptoms. In the intervention group, mean score of quality of life related to constipation at the end of intervention and the 10-day follow-up was 0.56 and 0.19 less than the control group (Decrease in the mean score of quality of life related to constipation indicates an improvement in the quality of life) (*P* < 0.0001).

**Conclusion:**

The results showed the positive effect of auricular acupressure on reducing the severity of constipation symptoms and improving the quality of life in old patients living in the residential care home. This non-pharmaceutical practice can be used by nurses as an inexpensive, safe, acceptable, and non-invasive nursing care for older people with constipation in homes, medical centers, or nursing homes.

## Introduction

According to the World Health Organization (WHO) report, the number of people aged 65 or older is projected to grow from an estimated 524 million in 2010 to nearly 1.5 billion in 2050, and more than 15% of the world's population will be older people [1]. In Iran, the growth of the old people population is very high in the short term, often due to the rapid decline in the birth rate. According to forecasts, Iran is the third country in the Middle East region in terms of the rapid growth of the old people population [2]. The number of Iranian older people is expected to double between 2016 and 2040 [3]. An increase in the old people population inevitably increases the prevalence of chronic diseases and disabilities [2].

Constipation is a common and significant health problem in the older people that has negative effects on their quality of life, increases economic costs, and can lead to psychological symptoms such as anxiety, stress and severe physical symptoms such as intestinal obstruction [4]. It is important to identify interventions to improve the quality of life of older people living in care homes (RCH), as they are one of the groups most at risk of poor quality of life in society [5]. The incidence of constipation among the older people is approximately 30%, and an even higher prevalence has been reported among the older people under long-term care in hospitals or RCH [6]. Up to 70% of people who live in the RCH, are susceptible to constipation. Chronic diseases and medication use are significantly associated with this constipation [7].

A quarter of patients with constipation do not achieve the desired treatment results [8]. About 85% of patients with constipation use laxatives [9]. However, repeated use of laxatives leads to malabsorption and interaction with other drugs and can even worsen constipation by changing the mucosa. Other side effects of laxatives that have been reported in the older people include dehydration, electrolyte imbalance, and hepatotoxicity [7]. Therefore, to reduce the side effects of constipation in older people, it is advantageous to use complementary interventions with lower side effects [10].

Some studies have reported that auricular acupressure (AA) as a non-pharmacological approach, has promising results in the management of constipation [11]. In AA, the auricular acupoint is stimulated by a small round object [12] (such as vaccaria seeds or magnetic pellets). As a non-invasive and non-acupuncture method, AA can be easily applied to the patients without pain and side effects [13]. Based on the meridian theories, acupoints stimulation can improve the flow of vital energy or (Qi, pronounced "chee") and blood, and these changes lead to experiencing a feeling of health, and create balance in the body systems [14]. There are different neurophysiological connections between the reflex acupoints of the external ear and the autonomic and central nervous systems. Accordingly, each anatomical unit of the body has a point of reflection on the surface of the external ear in each of the body organs [15]. Stimulation of specific acupoints directly stimulates the sympathetic and parasympathetic nerves. Therefore, diseases that affect the organs associated with these acupoints may be relieved by AA [16]. Seven acupoints that are probably effective in reducing constipation symptoms; include: large intestine, rectum, San Jiao, spleen, lung, sympathetic, and subcortex. The effect of AA in the management of constipation is thought to be through the regulation of gastrointestinal activity, blood circulation, and Qi flow; which probably leads to increased intestinal peristalsis, the distribution of body fluids to the intestine, and consequently, the desire to defecate [12]. Shin et al. (2018) designed AA for women with breast cancer undergoing chemotherapy and reported that AA significantly reduced symptoms of constipation and improved quality of life in patients undergoing chemotherapy [17]. Also, Li et al. (2014) reported a decrease in the constipation symptoms and improvement in the quality of life of older people that live in RCH after receiving AA with magnetic pellets. The use of magnetic pellets had side effects such as dizziness and the risk of affecting the normal function of the electrical device implanted in the body [12]. Meanwhile using vaccaria seeds alone for AA is a non-invasive, cheap and safe method. Vaccaria seeds have an anti-inflammatory effect (to prevent inflammation caused by the pressure of seeds on the ear or the irritating effect of plaster on the skin) and provides the possibility of applying pressure exactly at the desired acupoint [18]. However, most studies have been conducted in China; Due to geographic differences around the world, further studies are necessary to confirm the efficacy and safety of AA for constipation [19].

According to WHO, the most important problem that complementary and alternative medicine faces, is the lack of research information in this field [20]. It is recommended that nurses rigorously design, implement and evaluate the effects of new non-pharmacological interventions to improve constipation amongst older adults in long-term care settings [11]. Since there have been limited studies with different intervention methods on the effect of AA on constipation in the older people. Therefore, this study was performed to investigate the effect of auricular acupressure on constipation and related quality of life in the older people living in the residential care home.

## Hypothesis

Auricular acupressure has effects on the constipation and related quality of life in older people who live in the residential care home.

## Materials and method

### Design

This study was a randomized control clinical trial; and the intervention variable is auricular acupressure and the outcome variables include constipation symptoms and related quality of life among the older people. Participants were older people who are living in the four residential care homes in the southeast of Iran (in Kerman city). The study was conducted from January to April -2021.

### Sampling and participants

In this study, significance level (0.05) and statistical power (0.9) were used to determine the sample size (Z_1-α/2_ = 1.96, Z_1-β_ = 1.28). The minimally clinical important difference (MCID) in PAC-SYM score between the experimental and the control group in 10-day intervention, was μ_1_-μ_2_ = 0.5. Standard deviation (δ_1_ = 0.38, δ_2_= 0.53) were extracted from the previous studies [12]. The calculated sample size was 18 participants in each group. Finally, to increase the accuracy and power of the study and to compensate for sample dropouts, a total of 30 participants were recruited in each group.

Male participants were divided into two groups by simple and stratified random sampling by blind statistician. At first, male participants were selected by the researcher and coded and classified based on the severity of the symptoms of constipation and laxative use, and then stratified by the statistician who was blind to the participants. Through the production of random numbers, the participants were divided into two groups of intervention and control by simple randomization. Due to the lack of concurrent access to all women in the research community, because of the spread of the corona virus and the quarantine of some women's RCH, they were divided into two groups at consecutive time intervals based on a computer-generated 4-block random sequence (with R software) which was created by the statistician (an example of two block sequences: "BABA" "ABBA"). After completion of randomization, the intervention was performed without delay. In this study, the participants and the statistician who was responsible for random allocation and data analysis were blinded to the participants’ group assignments. Participants were blind to group assignment, and in both groups, auricular plasters with the similar appearance were used. Thus, the participants were not informed about the differences (seeds and stimulation) in the intervention. The researcher implementing the intervention (care provider) was not blinded.

Seventy-six older people suffering from constipation were identified according to the medical records and daily reports, and chronic constipation was confirmed by Rome IV diagnostic criteria. Then, among the eligible older people who understood the purpose of the study and agreed to participate (more than 95% of eligible participants consented to participate and three people refused to give consent), 30 people were selected for the intervention group and 30 people for the control group. Inclusion criteria included: (1) age between 65–85, (2) Constipation according to the diagnostic criteria of Rome IV; must have more than 2 of the following criteria during at least the past 3 months with onset of symptoms at least 6 months ago (for > 25% of defecations): straining, lumpy or hard stools, sensation of incomplete evacuation, sensation of anorectal obstruction/blockage, manual maneuvers to facilitate defecation, less than three spontaneous bowel movements per week [6], (3) Cognitive status with a score ≥ 6 in the abbreviated mental test and be able to report their bowel function. The abbreviated mental test score is a 10-point assessment to rapidly assess the cognition of older people patients for the possibility of dementia, screening delirium, and is used to identify any confusion [21]. Exclusion criteria included: (1) having local lesions/infection of the ears, or absence of ear [12], (2) having an abdominal operation in the past 6 months [17], (3) receive treatment for constipation (except laxatives) besides auricular acupressure [17], (4) increased laxative use (in order to reduce the possible effect of the confounding factor, increasing laxative consumption, on the outcome variables) [18], (5) having dementia or delirium disorder or suffering from major physical and mental illnesses affecting constipation [12], (6) if Participants do not follow the instructions in maintaining the auricular plasters in seven acupoints for more than one day.

## Data collection

Using a researcher made questionnaire, demographic and clinical characteristics of older people were collected before the intervention. This questionnaire consisted of two parts. The first part included participant demographic information and the second part contained the clinical information, including past medical history, regular drug use, duration of constipation, laxative use, number of defecations, daily intake of fruits and vegetables (taken from Iranian Mini Nutrition Assessment Questionnaire; it is a well-validated tool for assessing nutrition in old people and includes questions derived from general assessment, dietary, and unit daily intake of fruits, and vegetables) [22], physical activity (taken from International Physical Activity Questionnaires; this measure assesses the intensity of physical activity and sitting time that people do as part of their daily lives to estimate total physical activity in min/week) [23]. Patient's cognitive status score was based on Abbreviated Mental Test [21].

PAC-SYM (Patient Assessment of Constipation-Symptom) and PAC-QOL (Patient Assessment of Constipation-Quality of Life) questionnaires were used to measure constipation score and constipation related quality of life. The PAC-SYM and PAC-QOL questionnaires were completed before the intervention, at the end of the intervention, and at 10-day follow-up (Day 20; in order to evaluate the long-term effects of auricular acupressure after removing vaccaria seeds) [12] in the two groups of intervention and control by the older people or through the interview by the researcher. The PAC-SYM was developed by Frank et al. (1999). This questionnaire includes 12 items with three subscales (abdominal symptoms 4 items, rectal symptoms 3 items, and stool symptoms 5 items). Questions that assessed the severity of constipation symptoms were on a 5-point Likert scale; 0 (no problem) to 4 (very severe) and also the mean score of the questionnaire is from a minimum of 0 (no problem) to a maximum of 4 (very severe). The validity of this questionnaire was first confirmed by Frank et al. using Cronbach's alpha coefficient, the internal consistency of this questionnaire was 0.89 [24]. The Persian version of the PAC-SYM questionnaire has validity and reliability with a content validity index (CVI = 0.91) and Cronbach's alpha coefficient of 0.87 [25]. The PAC-QOL was developed by Marquis et al. (2005). This questionnaire has 28 questions with a 5-point Likert scale; 0 (never) to 4 (too much / always). The mean score of the questionnaire is at least 0 to maximum of 4 and higher scores indicate the more negative impact of constipation on various aspects of quality of life. As a result, higher scores indicate a decrease in overall quality of life and lower scores indicate improvement in overall quality of life. This questionnaire includes four subscales: physical discomfort (4items), psychosocial disorders (8 items), satisfaction (5 items), and worries and concerns (11 items). Marquis et al. (2005) confirmed the validity of this questionnaire. They reported the reliability of this questionnaire by Cronbach's alpha (0.93) and the intra-class correlation coefficient (ICC = 82.0) [26]. The Persian version of the PAC-QOL has good validity and reliability in chronic constipation and its reliability has been reported to be 0.92 based on Cronbach’s alpha [27].

### Intervention

Before applying AA, the surface of the ears was examined; if a foreign substance and/or oil were found in the ears, they were removed using alcohol swabs. At the beginning of the study, the amount and type of laxative use, the levels of physical activity, and the number of fruits and vegetables consumed by the patient were recorded. Participants should maintain usual dietary habits and levels of physical activity and laxative use.

**Fig. 1:**
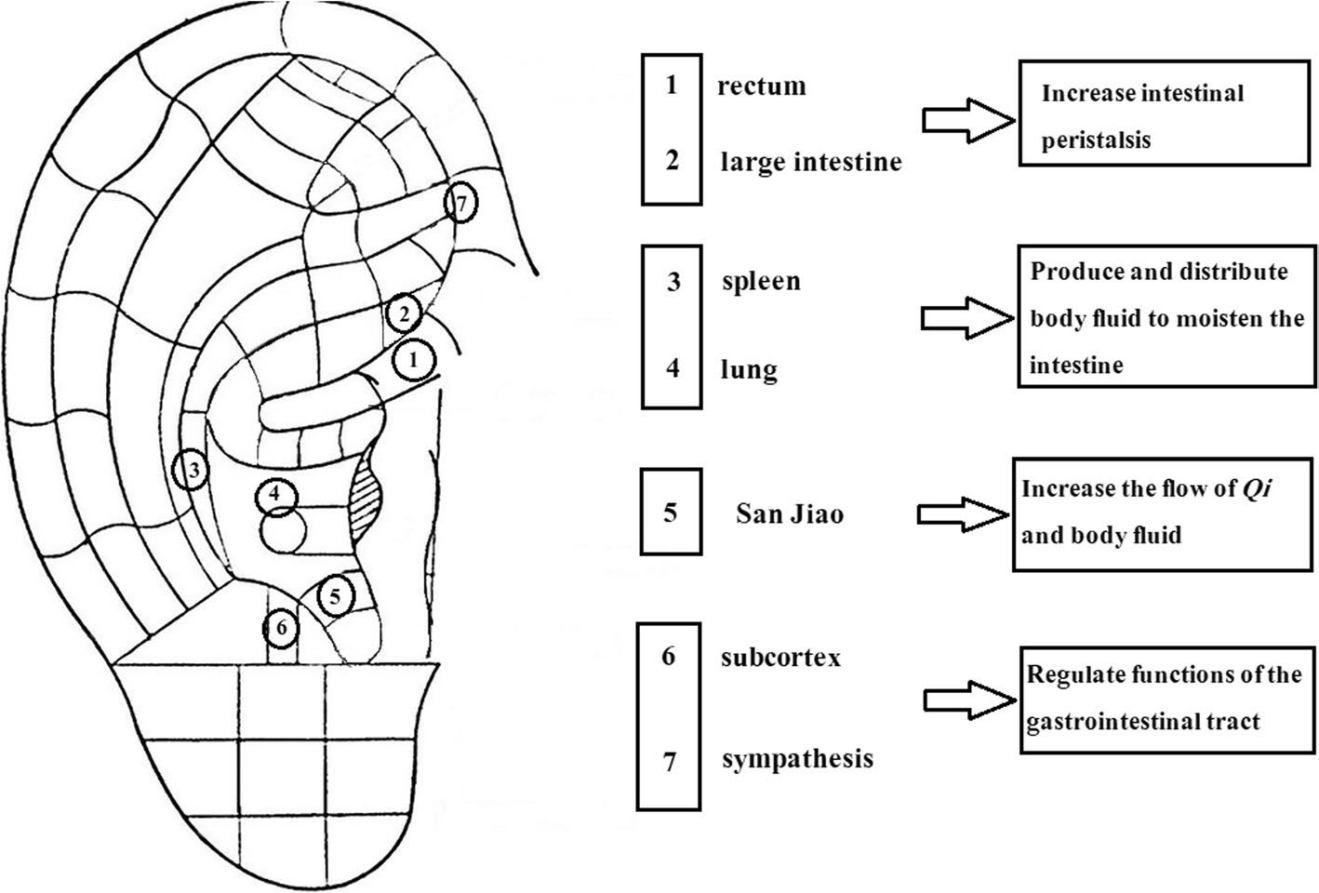
Seven auricular acupoints

The intervention was performed by one care provider who had passed the AA training course under the supervision of a specialist (research consultant) in traditional medicine and acupressure in a traditional medicine clinic. Course training included selecting the seven acupoints and taping the auricular plasters with vaccaria seeds. In order to investigate and increase the care provider adherence, the implementation of the intervention was reviewed daily by an acupressure specialist (research consultant) and other researchers. The intervention included treatment with AA on seven acupoints (large intestine, rectum, San Jiao, spleen, lung, sympathetic, and subcortex), and these points were effective in alleviating constipation symptoms (see Fig. 1). The acupoints and locations were confirmed by a specialist in traditional medicine and acupressure, and previous studies [12].

In the intervention group, to apply continuous AA, auricular plasters with vaccaria seeds were used and the researcher manually stimulated seeds three to four times a day until the participant felt a slight pain. To prevent interference between the intervention and control groups, as well as blinding the participants, seedless auricular plasters were used for the control group in the seven acupoints as sham acupressure and weren't actually caused the stimulation. In a systematic review and meta-analysis of randomized controlled trials regarding the efficacy of auriculotherapy for constipation in adults conducted by Yang et al. (2014), the duration of AA varied from 3 to 60 days [19]. In this study, based on the previous study [12] and with the consultation of a traditional and acupressure medicine specialist, the intervention was conducted for 10 days.

To prevent undue pressure on the two ears, auricular plasters (with seeds) were placed on one ear every five days, 5 days on the right ear, and after 5 days, Auricular plasters (with seed) were directly removed from the ear and in the second 5 days, new auricular plasters (with seeds) were placed on the left ear. If the auricular plasters were removed from the ear earlier than five days, the researcher again used the new auricular plasters. All participants were evaluated for side effects and no serious complications occurred among the participants. The only side effect was local itching, which was reported by 9 participants. Local itching was described as mild, transient and tolerable and only one participant dropped out in the intervention group due to local itching and unwillingness to continue the study.

### Statistical analysis

SPSS software version 22 was used for data analysis. Mean, standard deviation, frequency, and percentage were used to describe the demographic and clinical characteristics. At the baseline, the homogeneity of characteristics of the intervention and control group was assessed by chi-square test, Fisher’s exact test, and independent t-test. Also, repeated measure ANOVA and ANCOVA tests were used to compare the two groups in repeated measures in PAC-SYM and PAC-QOL. The data were analyzed using an intention-to-treat (IIT) method and missing data was estimated by EM (Expectation Maximization) algorithm. For each group, 30 participants were included in each analysis. The data were analyzed at the significance level of 0.05.

## Results

**Fig. 2:**
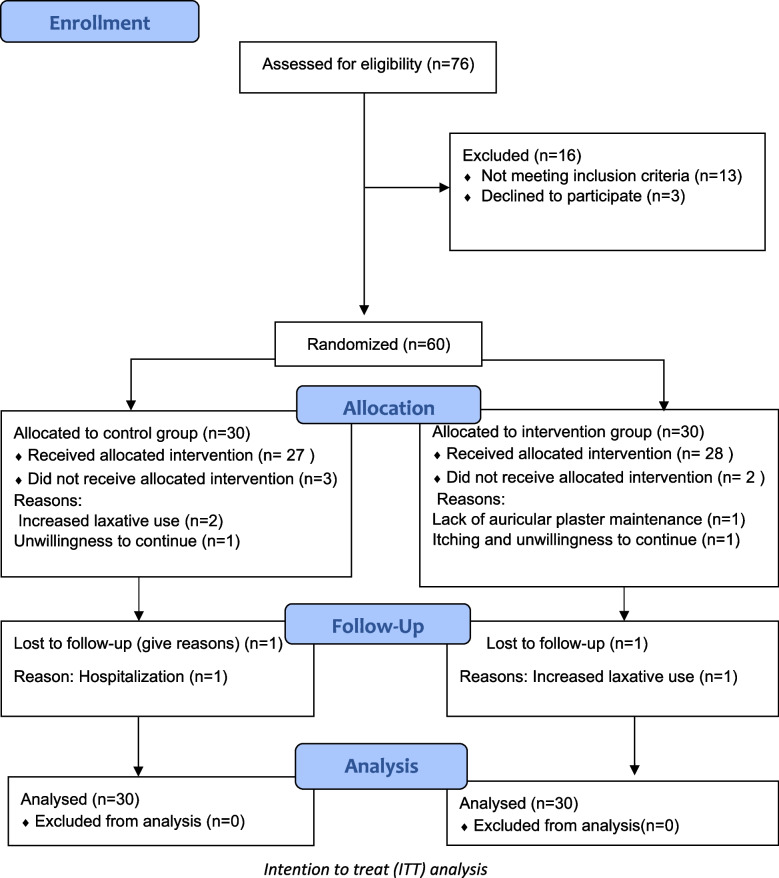
Diagram of selection of participants and allocated of groups

Ultimately, 53 participants completed the study, with an attrition rate of 11%. Overall, three participants dropped out in the intervention group due to itching caused by auricular plasters and unwillingness to continue, lack of auricular plaster maintenance, increased laxative use and four participants in the control group due to unwillingness to continue, increased laxative use, hospitalization (see Fig. 2). According to the Kolmogorov–Smirnov test, the data distribution was normal (*p* > 0.05).

The mean (standard deviation) age of all participants was 73.49(4.79) and ranged between 65–85 years old. The participants were 47% male and 53% female. According to the results, there was no significant difference between the two groups in terms of demographic and clinical characteristics (Tables 1 and 2). 58.5% of the participants had a low activity level and 30% of participants used laxatives regularly.

**Table 1:** Demographic characteristics (*N* = 60)

Variable		Intervention group (*n* = 30)	Control group (*n* = 30)	t ^1^	*p*
		Mean ± SD (range)	Mean ± SD (range)		
Age (years)		74.20 $\pm$ 5.37 (65–85)	72.77 $\pm$ 4.20 (66–84)	1.15	0.25
Duration of stay in RCH (year)		3.88 $\pm$ 1.60(1–6.5)	4.22 $\pm$ 1.91 (1–8)	-0.75	0.46
Characteristics		n (%)	n (%)	χ^2 2^	*p*
Gender	Male	14 (47)	14 (47)	0.00	1
	Female	16 (53)	16 (53)		
Educational level	Iilliterate	15 (50)	16 (53.3)		0.38 ^3^
	Primary or below	9 (30)	6 (20)		
	Lower secondary	3 (10)	7 (23.3)		
	Upper secondary	3 (10)	1 (3.3)		

^1^independent t-test

^2^chi-squared test

^3^Fisher’s exact test

**Table 2:** Clinical characteristics (*N* = 60)

Variable		Intervention group (*n* = 30)	Control group (*n* = 30)	t ^1^	*p*
		Mean ± SD (range)	Mean ± SD (range)		
Duration of constipation (years)		1.85 $\pm$ 1.04 (0.5–4)	2.15 $\pm$ 1.43 (0.5–6.5)	-0.92	0.36
Number of co-existing morbidities		1.60 $\pm$ 0.62 (1–3)	1.57 $\pm$ 0.93 (0–3)	0.16	0.87
Number of medications taken		4.17 $\pm$ 1.44 (0–6)	3.47 $\pm$ 1.78 (0–7)	1.67	0.1
Characteristics		n (%)	n (%)	χ2 ^2^	*p*
Activity	Little	16 (53.3)	19 (63.3)		0.76 ^3^
	Moderate	9 (30)	8 (26.7)		
	Much	5 (16.7)	3 (10)		
Daily Consumption of fruits and vegetables	Little	7 (23.3)	8 (26.7)	0.64	0.72
	Moderate	10 (33.3)	12 (40)		
	Much	13 (43.3)	10 (33.3)		
Number of defecations	Daily	12 (40)	11 (36.7)	0.29	0.86
	One time/2 days	10 (33.3)	12 (40)		
	One time/more than 3 days	8 (26.7)	7 (23.3)		
Hypertension	Yes	14 (46.7)	17 (56.6)	0.60	0.44
	No	16 (53.3)	13 (43.4)		
Heart disease	Yes	11 (36.6)	9 (30)	0.30	0.58
	No	19 (63.4)	21 (70)		
Diabetic mellitus	Yes	12 (40)	10 (33.3)	0.28	0.59
	No	18 (60)	20 (66.6)		
Neurological diseases	Yes	5 (16.6)	7 (23.3)	0.41	0.52
	No	25 (83.4)	23 (76.7)		
Psychiatric diseases	Yes	6 (20)	7 (23.3)	0.09	0.75
	No	24 (80)	23 (76.7)		
Use of laxatives	Yes	9 (30)	11 (36.6)	0.30	0.58
	No	21 (70)	19 (63.4)		
Regular use of medication	Yes	28 (93.3)	25 (83.3)		0.42 ^3^
	No	2 (6.7)	5 (16.7)		
Use of ARB^1^ / ACE^2^ and beta-blockers 1. Angiotensin receptor blockers 2. Angiotensin-Converting Enzyme	Yes	24 (80)	19 (63.4)	2.05	0.15
	No	6 (20)	11 (36.6)		
Use of diuretics	Yes	13 (43.3)	10 (33.3)	0.64	0.43
	No	17 (56.6)	20 (66.6)		
Use of calcium blockers	Yes	6 (20)	9 (30)	0.8	0.37
	No	24 (80)	21 (70)		
Use of calcium supplements	Yes	15 (50)	11 (36.7)	1.09	0.30
	No	15 (50)	19 (63.3)		
Use of antidepressants and benzodiazepines	Yes	13 (43.3)	16 (53.3)	0.6	0.44
	No	17 (56.6)	14 (46.7)		

^1^independent t-test

^2^chi-squared test

^3^Fisher’s exact test

### PAC-SYM (Constipation symptoms)

The mean scores of the PAC-SYM and its subscales and the mean difference between the two groups in the three-time points are presented in Table 3. There was no significant difference in the mean score of PAC-SYM and its subscales between the two groups before the intervention (*p* < 0.05). The difference between groups and times was significant in PAC-SYM scores. It was indicated that there was a significant difference between the intervention and control groups across all the follow up time points (Day 10 and 20) and the scores of the intervention group in PAC-SYM were significantly lower compared to the control group (F_(Group)_ = 52.75, *p* < 0.001, *η*_*p*_^*2*^ = *0.48*). The effect size with partial Eta squared greater than 40 indicates that the intervention had a large effect on the outcome variables. At the end of the intervention (Day 10), analysis of covariance (ANCOVA) test indicated a significant difference between the study groups; The intervention group's scores on the PAC-SYM and its subscales were significantly lower compared to the control group. The mean score PAC-SYM at the end of the intervention was 0.36 less in the intervention group than in the control group (F = 89.6, *p* < 0.0001). The difference between groups and times was significant in scores of abdominal, rectal, and stool symptoms (F = 30.46, F = 15.32, F = 36.06, respectively, *p* < 0.001). The difference in the mean scores of abdominal, rectal, and stool symptoms at the end of the intervention in the intervention group was 0.29, 0.24, and 0.51 less than the control group, respectively (*p* < 0.0001). A significant decrease in the PAC-SYM score and its subscales at the end of the intervention in the intervention group indicates a reduction in the severity of constipation symptoms and the positive effects of AA on the reduction of constipation.

**Table 3:** Comparison of PAC-SYM and its subscales between the intervention and the control groups (*N* = 60)

Variable	Groups	Before intervention Mean ± SD	End of intervention (Day 10) Mean ± SD	10-day follow-up (Day 20) Mean ± SD	Source	F^a^	*P*	*Partial Eta squared*
PAC-SYM	intervention group	1.31 ± 0.31	0.87 ± 0.29	1.11 ± 0.31	Time Group Time*Group	0.58 52.75 40.54	0.45 < 0.0001 < 0.0001	0.01 0.48 0.42
	Control group	1.37 ± 0.31	1.27 ± 0.29	1.25 ± 0.28				
	Β^b^(95% CI)	-0.05(-0.21,0.10)	-0.36(-0.43,-0.28)	-0.08(-0.16,-0.01)				
	F^c^	0.48	89.6	5.3				
	*P*	0.49	< 0.0001	0.025				
Abdominal symptoms	intervention group	0.72 ± 0.31	0.32 ± 0.26	0.49 ± 0.33	Time Group Time*Group	0.02 13.66 30.46	0.88 0.001 < 0.0001	0.00 0.19 0.35
	Control group	0.66 ± 0.30	0.57 ± 0.34	0.50 ± 0.34				
	Β^b^(95% CI)	0.06(-0.1, 0.22)	-0.29(-0.39, -0.20)	-0.06(-0.17, 0.05)				
	F^c^	0.55	36.97	1.12				
	*P*	0.46	< 0.0001	0.28				
Rectal symptoms	intervention group	1.04 ± 0.34	0.75 ± 0.29	1.00 ± 0.39	Time Group Time*Group	3.95 2.51 15.32	0.05 0.12 0.001	0.06 0.04 0.21
	Control group	1.16 ± 0.33	1.07 ± 0.32	1.02 ± 0.33				
	Β^b^(95% CI)	-0.12(-0.30, 0.03)	-0.24(-0.35, -0.13)	0.05(-0.12, 0.22)				
	F^c^	2	19.62	0.31				
	*P*	0.16	< 0.0001	0.58				
Stool symptoms	intervention group	1.96 ± 0.51	1.40 ± 0.40	1.77 ± 0.46	Time Group Time*Group	0.24 30.47 36.06	0.63 < 0.0001 < 0.0001	0.004 0.35 0.39
	Control group	2.03 ± 0.43	1.95 ± 0.42	1.96 ± 0.41				
	Β^b^(95% CI)	-0.07(-0.32, 0.17)	-0.51(-0.65, -0.37)	-0.14(-0.26, -0.02)				
	F^c^	0.36	51.26	5.15				
	*P*	0.55	< 0.0001	0.02				

^a^repeated measure ANOVA

^b^Mean Difference

^c^ANCOVA

To evaluate the persistence of the long-term effects of AA, in 10-day follow-up (Day 20) results indicated that, despite the increase in the PAC-SYM score and its subscale in the intervention group, there was still a significant difference (with ANCOVA test) in the mean PAC-SYM score between the intervention and control groups (F = 5.3, *P* = 0.025). The PAC-SYM mean score in the intervention group was 0.08 less than the control group. In 10-day follow-up, ANCOVA test indicated that only in stool symptoms subscale was a significant difference observed between the intervention and control groups, and no significant differences were observed in the abdominal and rectal symptoms. The stool symptoms mean score in the intervention group was 0.14 less than the control group (F = 5.15, *P* = 0.02). A significant decrease in the score of PAC-SYM and stool symptoms subscale at the 10-day follow-up in the intervention group indicates the persistence of the long-term positive effects of AA on the reduction of stool symptoms associated with constipation.

In the comparison of the mean score of PAC-SYM separately in the intervention and control groups at three time points (Table 4), the results of the repeated measure ANOVA test showed that there was a significant difference between the mean score of PAC-SYM in the intervention group at the three time points (*P* < 0.0001). The lowest mean score of PAC-SYM in the intervention group was 0.87 (0.29) at the end of the intervention, which indicated the peak effect of AA with vaccaria seeds in reducing the severity of constipation symptoms at the time of completion of the intervention. Also, in the control group, there was a significant difference between the mean score of PAC-SYM at three time points (*P* < 0.0001). At the end of the intervention, the mean score of constipation in the control group was 1.27 (0.29). Probably, this slight decrease in the mean score of constipation at the end of the intervention compared to before the intervention was related to the use of sham AA.

**Table 4:** Comparison the mean score of PAC-SYM separately in the intervention and control groups at three time points

Group	Before intervention Mean ± SD	End of intervention (Day 10) Mean ± SD	10-day follow-up (Day 20) Mean ± SD	*F*^*a*^	*P*
Intervention	1.31 ± 0.31	0.87 ± 0.29	1.11 ± 0.31	157.46	< 0.0001
Control	1.37 ± 0.28	1.27 ± 0.27	1.25 ± 0.28	20.25	< 0.0001

^a^ANCOVA

### PAC-QOL (Patient Assessment of Constipation-Quality of Life)

The mean scores of the PAC-QOL and its subscales and mean difference between two groups in the three-time points are summarized in Table 5. The mean (SD) satisfaction subscale before intervention was 2.77(0.33); satisfaction subscale range is 0–4, higher scores indicate patient dissatisfaction with their current condition and treatment. There was no significant difference in the mean score of PAC-QOL and its subscales between the two groups before the intervention (*p* < 0.05), but after intervention, the difference between groups and times was significant in PAC-QOL scores. A significant difference was indicated between the intervention and control groups across all the follow up time points (Day 10 and 20) and the scores of the intervention group in PAC-QOL were significantly lower compared to the control group (F_(Group)_ = 64.85, *p* < 0.001,* η*_*p*_^*2*^ = *0.79*). The effect size with partial Eta squared greater than 40 indicates that the intervention had a large effect on the outcome variables.

**Table 5:** Comparison of PAC-QOL and its subscales between the intervention and the control groups (*N* = 60)

Variable	Groups	Before intervention Mean ± SD	End of intervention (Day 10) Mean ± SD	10-day follow-up (Day 20) Mean ± SD	Source	F^a^	*P*	*Partial Eta squared*
PAC-QOL	intervention group	1.76 ± 0.36	1.12 ± 0.34	1.43 ± 0.38	Time Group Time*group	0.29 64.85 48.90	0.59 < 0.0001 < 0.0001	0.005 0.79 0.46
	Control group	1.84 ± 0.34	1.68 ± 0.33	1.65 ± 0.33				
	Β^b^(95% CI)	-0.08(-0.26, 0.10)	-0.49(-0.60, -0.39)	-0.15(-0.24, -0.07)				
	F^c^	0.75	93.32	12.67				
	*P*	0.39	< 0.0001	0.001				
Physical discomforts	intervention group	2.24 ± 0.63	1.12 ± 0.57	1.92 ± 0.69	Time Group Time*group	0.60 34.62 55.26	0.44 < 0.0001 < 0.0001	0.01 0.38 0.49
	Control group	2.42 ± 0.56	2.21 ± 0.57	2.21 ± 0.57				
	Β^b^(95% CI)	-0.17(-0.48, 0.13)	-0.98(-1.20, -0.76)	-0.16(-0.38, 0.08)				
	F^c^	1.30	82.63	1.82				
	*P*	0.26	< 0.0001	0.18				
Psychosocial discomforts	intervention group	0.91 ± 0.24	0.62 ± 0.20	0.76 ± 0.23	Time Group Time*group	0.02 8.58 23.90	0.89 0.005 < 0.0001	0.00 0.13 0.30
	Control group	0.95 ± 0.26	0.82 ± 0.21	0.80 ± 0.20				
	Β^b^(95% CI)	-0.05(-0.17, 0.08)	-0.17(-0.24, -0.10)	-0.004(-0.07, 0.07)				
	F^c^	0.51	25	0.01				
	*P*	0.48	< 0.0001	0.92				
Worries and concerns	intervention group	1.81 ± 0.45	1.11 ± 0.37	1.43 ± 0.42	Time Group Time*group	0.35 53.07 33.5	0.55 < 0.0001 < 0.0001	0.006 0.48 0.37
	Control group	1.89 ± 0.41	1.72 ± 0.42	1.70 ± 0.47				
	Β^b^(95% CI)	-0.07(-0.29, 0.15)	-0.56(-0.68, -0.44)	-0.21(-0.33, -0.09)				
	F^c^	0.44	88.92	11.29				
	*P*	0.51	< 0.0001	0.001				
Satisfaction	intervention group	2.67 ± 0.30	1.95 ± 0.39	2.22 ± 0.34	Time Group Time*group	0.04 60 24	0.84 < 0.0001 < 0.0001	0.001 0.51 0.30
	Control group	2.77 ± 0.37	2.62 ± 0.37	2.62 ± 0.38				
	Β^b^(95% CI)	-0.11(-0.28, 0.07)	-0.60(-0.74, -0.45)	-0.31(-0.43, -0.19)				
	F^c^	1.50	71.23	27.14				
	*P*	0.22	< 0.0001	< 0.0001				

^a^repeated measure ANOVA

^b^Mean Difference

^c^ANCOVA

At the end of the intervention (Day 10), ANCOVA test indicated a significant difference between the study groups; The intervention group's scores on the PAC-QOL and its subscales were significantly lower compared to the control group. In the intervention group, mean score of PAC-QOL at the end of the intervention was 0.49 less than the control group (F = 93.32, *p* < 0.0001). The difference between groups and times was significant in scores of physical discomforts, psychosocial discomforts, worries and concerns, and satisfaction (F = 55.26, F = 23.90, F = 33.5, F = 24, respectively, *p* < 0.0001). In the intervention group, the difference in the mean scores of physical discomforts, psychosocial discomforts, worries and concerns, and satisfaction at the end of the intervention was 0.98, 0.17, 0.56, and 0.60 less than the control group, respectively (*p* < 0.0001). A significant decrease the PAC-QOL score and its subscales indicates a reduction in the negative impact of constipation on the quality of life of patients and improves the overall quality of life and the positive effects of AA on improving quality of life in intervention group.

ANCOVA test indicated that there was a significant difference between the two groups in the mean score of PAC-QOL and subscales of worries and concerns and satisfaction in 10-day follow-up (Day 20) (*P* < 0.001). The difference in the mean scores of PAC-QOL, worries and concerns, and satisfaction in the intervention group was 0.15, 0.21, and 0.31 less than the control group, respectively. Comparison of the two groups in the 10-day follow-up indicated that the positive effects of AA on improving the quality of life and subscales of satisfaction and reducing worries and concerns continue for a long time in the intervention group.

In the comparison of the mean score of PAC-QOL separately in the intervention and control groups at three time points (Table 6), the results of the repeated measure ANOVA test showed that there was a significant difference between the mean score of PAC-QOL in the intervention group at the three time points (*P* < 0.0001). The lowest mean score of PAC-QOL in the intervention group was 1.12 (0.34) at the end of the intervention, which indicated the effect of AA with vaccaria seeds in improvement the quality of life at the end of intervention. Also, in the control group, there was a significant difference between the mean score of PAC-QOL at three time points (*P* < 0.0001). At the end of the intervention, the mean score of PAC-QOL in the control group was 1.68 (0.33). There was a slight improvement in the quality of life at the end of the intervention compared to before the intervention, which was probably related to the use of sham AA in the control group.

**Table 6:** Comparison the mean score of PAC-QOL separately in the intervention and control groups at three time points

Group	Before intervention Mean ± SD	End of intervention (Day 10) Mean ± SD	10-day follow-up (Day 20) Mean ± SD	*F*^*a*^	*P*
Intervention	1.76 ± 0.36	1.12 ± 0.34	1.43 ± 0.38	142.59	< 0.0001
Control	1.84 ± 0.34	1.68 ± 0.33	1.65 ± 0.33	25.67	< 0.0001

^a^ANCOVA

## Discussion

This study was the first study with the new method (using vaccaria seeds for AA in seven specific acupoint) that confirmed the effect of AA on constipation and related quality of life among the older people in RCH.

### Clinical characteristics of participants

In this study, more than half of the older people had low levels of physical activity and indicated that insufficient physical activity was associated with an increased risk of constipation. Similar to a previous study that reported in the older people living in RCH, physical activity was significantly lower in the older people with constipation than the older people without constipation [28]. In this study, one-third of participants used laxatives regularly and still had constipation, the Rome IV diagnostic criteria indicated that laxative use was not effective in relieving constipation symptoms in some participants. In another study, 44% of participants reported that laxatives were not effective in relieving constipation, 60% reported that laxatives were not effective in relieving multiple symptoms of constipation, and 56% were dissatisfied with the treatment of constipation with laxatives. This indicates that laxatives are not effective for all patients and are not able to improve the quality of life of all patients with constipation [29]. The results of the satisfaction subscale of this study before the intervention showed that the participants had little to moderate satisfaction with their current condition and treatment. Similarly, a study reported that many patients were dissatisfied with current constipation treatments due to ineffectiveness and side effects of medications, and that complementary treatment options would be beneficial for patients [30].

### Clinical effect of AA with vaccaria seeds in reducing constipation symptoms

The results of this study showed that participants who received AA with vaccaria seeds on seven acupoints (large intestine, rectum, San Jiao, spleen, lung, sympathetic, and subcortex) had significantly lower PAC-SYM and abdominal, rectal, and stool symptoms scores. Therefore, AA with vaccaria seeds is a promising non-pharmacological intervention to reduce the severity of constipation and abdominal, rectal, and stool symptoms among the older people in RCH. The results of the follow-up period showed that AA with vaccaria seeds has significant long-term effects in reducing the symptoms of constipation and stool, but it did not have a significant effect on abdominal and rectal symptoms, which is probably due to the short duration of the intervention and the application of AA only on one ear every five days. Probably, the simultaneous application of AA on both ears has a stronger effect. According to the results, the hypothesis of the research about the effect of AA on constipation among the older people in RCH is accepted.

Similar to these results, Li et al.’s (2014) study reported alleviated constipation symptoms after receiving AA for 10 days, using magnet pellets in intervention group at the same seven auricular acupoints on the older people living in RCH; moreover, a significant improvement in stool symptoms subscale was observed among participants who received AA [12]. However, in the study by Li et al. (2012), AA performed using vaccaria seeds and magnetic pellets on seven acupoints similar to this study for 21 days in older people, reported that AA had no significant effect on PAC-SYM scores. Because in the study of Li et al. (2012), the sample size was small, so it was not enough to confirm the effects of AA [31]. Yang et al. (2017) investigating Clinical efficacy of acupuncture plus auricular plaster therapy vs acupuncture alone in the treatment of constipation in old patients, reported improvement in constipation symptoms in the AA group was better, and the therapeutic effect of acupuncture with AA is better than acupuncture alone in elderly patients with constipation  [32]. To investigate the effects of AA on constipation in other patients, Lahijanian et al. (2020), investigating the effect of AA with vaccaria seeds on constipation in hemodialysis patients, showed that AA is effective at reducing the severity of constipation in hemodialysis patients [18]. In the Lahijanian study, two acupoints (large intestine and rectum) were the same as the present study. Similar to the results of the present study that indicate the persistence of the long-term positive effects of AA on the reduction of stool symptoms associated with constipation, Lahijanian's study reported that AA has stable effects on the reduction of constipation. Shin et al. (2018) designed AA using vaccaria seeds for women with breast cancer receiving chemotherapy for 6 weeks to relieve constipation symptoms. Similar to the present study they reported that AA significantly reduced constipation symptoms in patients undergoing chemotherapy [17]. Although various constipation assessment tools were used in the study of Shin et al. (2018), the same seven acupuncture points were used. Therefore, this study showed that stimulation of these seven acupoints is effective in reducing constipation symptoms. The study by Yang et al. (2014) investigating the effectiveness of AA in patients with opium-induced constipation reported that AA was not significantly different in constipation symptoms [33]. While regarding the symptoms of constipation, it was inconsistent with the findings of the present study, and the reason for the difference is probably in the research population and the etiology of constipation caused by opium (unlike the present study, which is the cause of constipation caused by the pathophysiology of old age) and the tool used to collect information about constipation symptoms. Min et al. (2020) investigated the effects of AA on functional constipation in female students for 6 weeks. The results showed that the severity of constipation improved significantly in the group undergoing AA [34]. In the Min study, five acupoints were selected for AA, and the selection of four acupoints, including the rectum, colon, lung, and San Jiao, was similar to this study. In a systematic review study conducted by Yang et al. (2014) to evaluate the effect of AA on adult constipation, after reviewing 15 clinical trials, the results showed that AA has a significant effect on constipation management [19].

### The effect of AA with vaccaria seeds in improving the constipation-related quality of life

The results of this study showed, similar to PAC-SYM results, that participants in AA group had significantly lower PAC-QOL and Physical discomforts, Psychosocial discomforts, Worries and concerns, and Satisfaction scores. Therefore, AA with vaccaria seeds is a promising intervention in improving the quality of life and its various dimensions, including the reduction of physical, psychosocial discomforts and increased satisfaction with treatment among the older people in RCH. The results of the follow-up period showed that AA with vaccaria seeds has significant long-term effects in improving the quality of life and also, showed that AA can reduce worries and concerns related to constipation for a longer period and AA can lead to increased satisfaction of patients. But it did not have a significant effect on Physical and Psychosocial discomforts, which is probably due to the short duration of the intervention. According to the results, the hypothesis of the research about the effect of AA on the constipation-related quality of life among the older people in RCH is accepted.

Similar to these results, the study of Li et al. (2014) reported an improvement in quality of life after receiving AA with magnet pellets for 10 days on the older people living in RCH, and also a significant improvement in satisfaction subscale, was observed among participants who received AA [12]. In another study conducted by Zhou et al. (2012) on the treatment of constipation in the older people with AA, vaccaria seeds were used for the intervention group for 50 days in eight acupoints (intestine, subcortex, and San Jiao same as in this study). It was reported, the constipation-related quality of life (PAC-QOL) significantly improved in the intervention group [35]. However, in the study by Li et al. (2012), AA was performed using vaccaria seeds and magnetic pellets on seven acupoints similar to this study for 21 days in older people, reported that AA had no significant PAC-QOL scores. Because in the study of Li et al. (2012), the sample size was small, so it was not enough to confirm the effects of AA [31]. Similar to the present study, the results of Shin et al.’s (2018) study showed that AA significantly improved the quality of life (PAC-QOL) in patients undergoing chemotherapy [17]. In another review study, Chen et al. (2018) reviewed 5 clinical trials that used AA in the treatment and prevention of constipation in leukemia patients undergoing chemotherapy. They reported AA can improve constipation and its associated quality of life [36]. Similar to present study, the study by Yang et al. (2014) investigating the effectiveness of AA in patients with opium-induced constipation reported that AA had a positive effect on constipation-related quality of life [33]. Min et al. (2020) investigated the effects of AA on functional constipation in female students for 6 weeks. The results showed that the constipation-related quality of life improved significantly in the group undergoing AA [34].

In this study, using vaccaria seeds for AA was approved as a safe and acceptable intervention in older people. The only side effect, local itching, was reported by participants due to auricular plaster (*n* = 9), local itching was described as mild, transient, and tolerable. One participant dropped out in the intervention group due to local itching and unwillingness to continue the study. Other studies did not report side effects of AA. Only the study of Li et al., (2014) reported that there were no serious side effects from using AA with magnetic pellet in older people and mild itching and dizziness were described [12]. Probably the cause of the dizziness was due to the use of a magnetic pellet.

## Conclusions

The results of this study show that using AA as a complementary medicine in recommended acupoints can relieve the symptoms of constipation without causing side effects and improve the quality of life associated with constipation, increase patients' satisfaction with their treatment, and decrease worries and concerns in the older people living in RCH. AA as a non-pharmacological intervention, reduced the patients' constipation and minimized the side effects of drug therapy and increased the quality of care for the older people. AA is easy, accessible, non-invasive, and has almost no side effects, and little space was needed to do it; these factors show that AA is comfortable and effective compared to other complementary and alternative medicine treatments. The training and clinical application of AA should be considered as a way to promote the physical health of elderly patients.

It is recommended that studies be conducted with a larger sample size and longer intervention period to evaluate the stability of the therapeutic effects of AA; also, studies be performed to compare the therapeutic effect of AA with oral laxatives on constipation and its quality of life in the elderly population.

## Limitations

The small sample size is due to the limitations caused by the spread of COVID-19 and the unavailability of all older people to enter the study due to the quarantine of some RCHs, as well as not including older people over 85 years old in the study, because the majority of this population living in RCH had low cognitive status in Iran. These factors can affect the generalizability and readers should pay attention to the sample size when reading the results of this study. The researcher implementing the intervention was not blinded and the influence of this subject on the results can be unknown.

## Data Availability

Authors make all datasets available to editors and reviewers, for the datasets, contact the corresponding author.
